# An explanation of the mystifying bakanae disease narrative for tomorrow's rice

**DOI:** 10.3389/fmicb.2023.1153437

**Published:** 2023-04-18

**Authors:** Qaiser Shakeel, Mustansar Mubeen, Muhammad Aamir Sohail, Sajjad Ali, Yasir Iftikhar, Rabia Tahir Bajwa, Muhammad Anjum Aqueel, Sudhir K. Upadhyay, Praveen Kumar Divvela, Lei Zhou

**Affiliations:** ^1^State Key Laboratory for Managing Biotic and Chemical Threats to the Quality and Safety of Agro-products, Institute of Agro-product Safety and Nutrition, Zhejiang Academy of Agricultural Sciences, Hangzhou, China; ^2^Cholistan Institute of Desert Studies, The Islamia University of Bahawalpur, Bahawalpur, Pakistan; ^3^Department of Plant Pathology, College of Agriculture, University of Sargodha, Sargodha, Pakistan; ^4^College of Plant Science and Technology, Huazhong Agricultural University, Wuhan, China; ^5^Department of Entomology, The Islamia University of Bahawalpur, Bahawalpur, Pakistan; ^6^Department of Environmental Science, VBS Purvanchal University, Jaunpur, Uttar Pradesh, India; ^7^Contec Global Agro Limited, Abuja, Nigeria

**Keywords:** rice, bakanae disease, mystifying, disease mechanism, management

## Abstract

Rice production is severely hampered by the bakanae disease (*Fusarium fujikuroi*), formerly recognized as *Fusarium moniliforme. F. moniliforme* was called the *F. fujikuroi* species complex (FFSC) because it was later discovered that it had some separate species. The FFSC's constituents are also well recognized for producing phytohormones, which include auxins, cytokinin, and gibberellins (GAs). The normal symptoms of bakanae disease in rice are exacerbated by GAs. The members of the FFSC are responsible for the production of fumonisin (FUM), fusarins, fusaric acid, moniliformin, and beauvericin. These are harmful to both human and animal health. This disease is common around the world and causes significant yield losses. Numerous secondary metabolites, including the plant hormone gibberellin, which causes classic bakanae symptoms, are produced by *F. fujikuroi*. The strategies for managing bakanae, including the utilization of host resistance, chemical compounds, biocontrol agents, natural goods, and physical approaches, have been reviewed in this study. Bakanae disease is still not entirely preventable, despite the adoption of many different tactics that have been used to manage it. The benefits and drawbacks of these diverse approaches are discussed by the authors. The mechanisms of action of the main fungicides as well as the strategies for resistance to them are outlined. The information compiled in this study will contribute to a better understanding of the bakanae disease and the development of a more effective management plan for it.

## 1. Introduction

**Figure 1:**
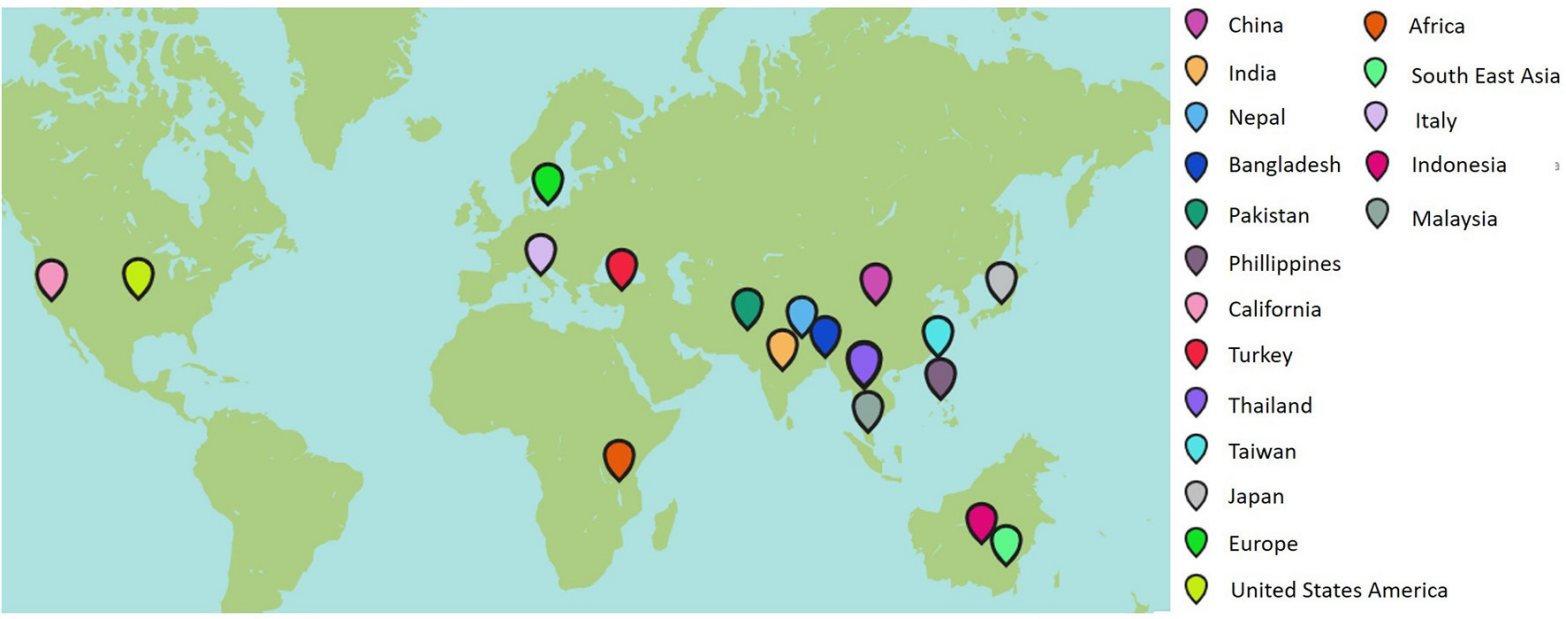
Geographical distribution of the bakanae disease.

Rice is one of the world's most vital staple food crops that is cultivated in diverse environmental conditions and is therefore subjected to various biotic and abiotic stresses. Insect pests and infections due to viruses, fungi, bacteria, and nematodes are the most significant biotic stresses impacting rice production. Bakanae (also known as foot rot) has emerged as a disease of serious concern among the critically valuable diseases of contemporary significance (Bashyal et al., 2016). It has been reported that one or more *Fusarium* species are involved in causing bakanae disease in rice. Though the bakanae pathogen is seed-borne in nature, it can cause infection at any stage of crop life from pre-emergence to maturation, leading to withering or poor germination of rice seeds (Iqbal et al., 2011). *F. fujikuroi* is one of the Asian clade members that has been studied extensively as a causal organism of the bakanae disease of rice. Generally, yield loss can be ~10–20% due to the disease, but it can also reach more than 70% under severe infection (Fiyaz et al., 2014). Bakanae disease is widespread in Asia and episodic in other rice-producing regions. The geographic distribution of the disease can be observed in Figure 1. “Bakanae” is a Japanese term that means “bad,” “kinky,” or “stupid” seedling, referring to the infrequent early seedling elongation caused by the production of mycotoxin (gibberellin) during the infection cycle (Fiyaz et al., 2016; Lee et al., 2018). The pathogen can infect a broad range of plant hosts and can be found all over the world. When a plant is fully grown, it can get infected anywhere from the roots to the crown to the stems to the leaf sheaths to the panicles. After initially being identified as *Lisea fujikuroi* Sawada in 1919, the fungus was renamed *Gibberella fujikuroi* (teleomorph) in 1931 (Ito and Kimura, 1931). *Fusarium moniliforme* was identified as its anamorph stage, but it is currently known as *Fusarium fujikuroi* (Sun and Snyder, 1981). The infection in rice plants can occur even after they have been transplanted, leading to stunted growth, poor tillering, and a lack of grain fill (Fiyaz et al., 2016).

Tall, gangly, and fewer tillers with yellowish green flag leaves, sterility, and grain discoloration are the characteristic symptoms of bakanae disease. Typically, infected plants die at later stages, whereas the panicles of surviving plants become impotent to produce grains and tolerate only unfilled panicles (Lee et al., 2018, 2022), ultimately reducing the crop yield. The source of secondary infection is through infected plants or seeds that disseminate by water or wind. The low survival rate of infected plants and high sterility of spikelets can contribute to yield losses reaching up to 50% in Japan, 40% in Nepal, 28.8% in Korea, 6.7–58.0% in Pakistan, and 3.0–95% in India (Lee et al., 2022). Taking into account the economic importance of rice and the dire threat posed by bakanae to rice production and yield, this study reviewed and summarized all important information about the pathogen from disease mechanisms to various control methods.

## 2. Associated species

**Figure 2:**
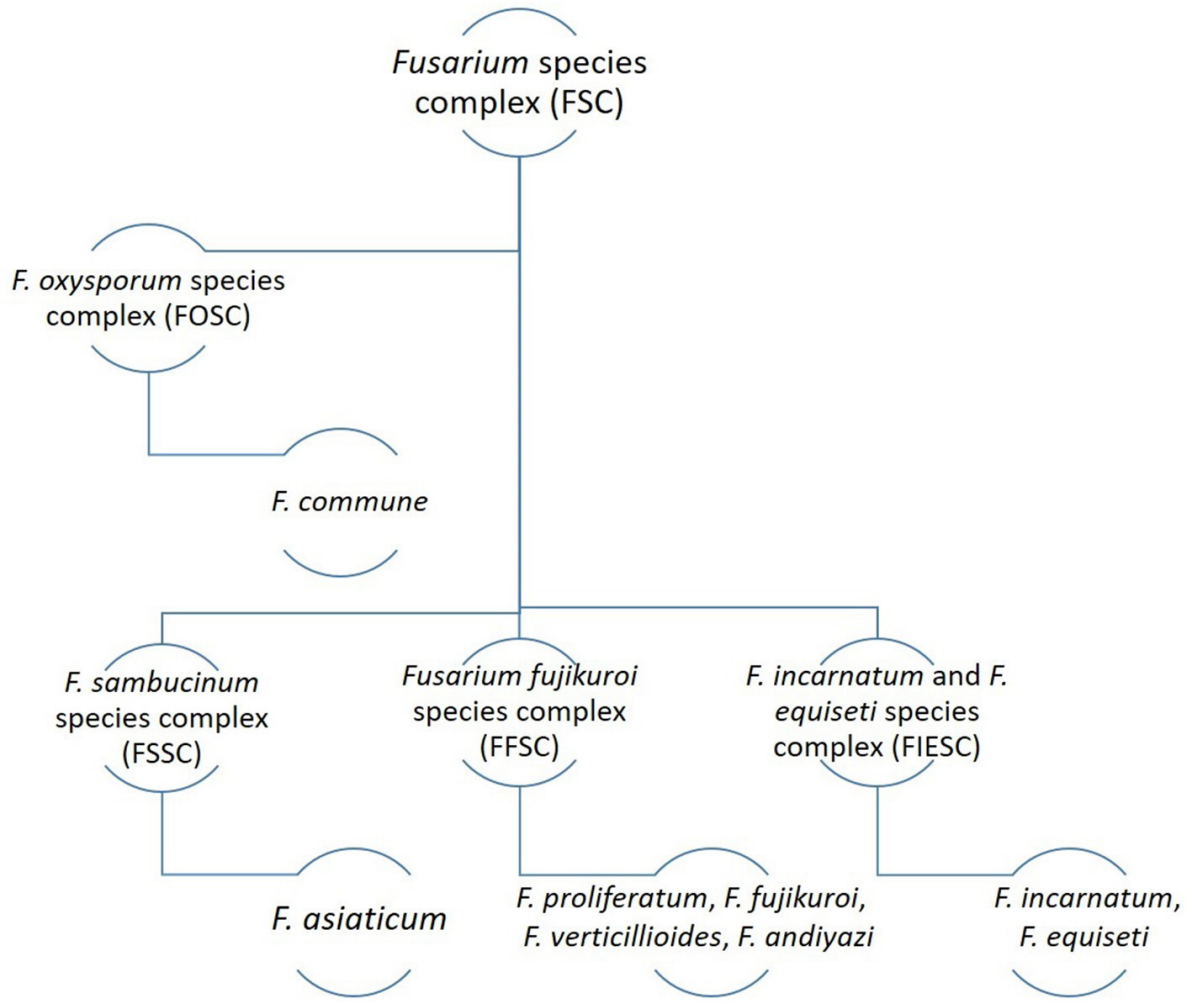
Fusarium species associated with the bakanae disease of rice.

Various species complexes of Fusarium have been reported to be associated with the bakanae disease of rice (Figure 2). Among them, four species of the *F. fujikuroi* species complex (FFSC), including *F. proliferatum, F. fujikuroi, F. verticillioides*, and *F. andiyazi*, are particularly responsible for causing bakanae disease of rice. Other associated species such as *F. commune* belonging to the *F. oxysporum* species complex (FOSC); *F. asiaticum* belonging to the *F. sambucinum* species complex (FSSC); and *F. incarnatum* belonging to the *F. equiseti* species complex (FIESC) have been isolated from rice seeds in different countries (Jahan et al., 2013; Choi et al., 2018; Avila et al., 2019; Jiang et al., 2020; Lee et al., 2022). Although members of FFSC are associated with the bakanae disease, *F. fujikuroi* is considered the fundamental species responsible for the characteristic symptoms (Jiang et al., 2020). Fusarium species' propensity for isolation and variation on rice seeds is dependent on the location and variations of those seeds, highlighting their variety. However, differences in the composition of the target species in different studies may be attributable to a number of factors, such as the target organ of the plant, sample size, geographical distribution of the pathogen species, and the effect of climate on species distribution (Moreira et al., 2020). Among all *Fusarium* spp. associated with the bakanae disease of rice, *F. fujikuroi* is the most dominant one having the highest isolation frequency. According to a study conducted in China, the *Fusarium* spp. isolated from three provinces showed the highest frequency of *F. fujikuroi* (80.05%), followed by *F. proliferatum, F. equiseti, F. incarnatum, F. commune, F. andiyazi*, and *F. asiaticum* with a frequency 8.31%, 5.94%, 2.61%, 1.66%, 0.95%, and 0.48%, respectively (Jiang et al., 2020).

## 3. Morphological characterization

Fusarium isolates that produce relatively slender macroconidia lacking momentous curvature but do not produce chlamydospores are categorized as FFSC. *F. fujikuroi* produces copious ovate or club-shaped microconidia in shorter chains (false heads) from monophilalides and monophialides with a flattened base and comprises zero to one septum (Jiang et al., 2020) and medium-length macroconidia that are slightly slender and lack substantial curvature. Additionally, three to five septate, tapered apical cells and poor development of basal cells is also a characteristic feature of macroconidia produced by *F. fujikuroi*. In *F. proliferatum*, copious club-shaped microconidia in false heads and chains are produced from monophialides and polyphialides and lack septation. However, macroconidia produced by *F. proliferatum* are slightly straight and slender and comprise three to five septations, curved apical cells, and poor development of basal cells. In the case of *F. andiyazi*, clavate to ovate microconidia having a flattened base are copiously produced in long chains lacking septation from monophialides, and macroconidia produced by this species are straight to partly curved comprising three to six septations (mainly three septations) with a slightly curved apical cell and a pedicellate basal cell. The formation of pseudochlamydospores is also a characteristic feature of macroconidia produced by *F. andiyazi* (Jiang et al., 2020). In FFSC, the morphological characteristics of *F. fujikuroi* and *F. proliferatum* are very similar, and a close relationship between these two species is also evident phylogenetically. Moreover, *F. andiyazi* and *F. verticillioides* exhibit similar morphology with the only difference being in the production of pseudo-chlamydospores by the former species, evident only in FFSC. Both these species share a close relationship phylogenetically as well (Jiang et al., 2020).

Fusarium isolates producing macroconidia with a conspicuous dorsiventral curvature or crescentiform are categorized as FIESC (Avila et al., 2019; Jiang et al., 2020). *F. equiseti* lacks microconidia production on PDA, while macroconidia are produced from sporodochia with a prominent dorsiventral curvature and needle-like apical cells are formed, which are more rounded as in *F. compactum*. *F. semitectum* (formerly known as *F. incarnatum*) produces copious mesoconidia (conidia produced in the aerial mycelia with the appearance of “rabbit ears” instead of sporodochia) from polyphialides that are spindle-shaped and straight with three to four septations (Jiang et al., 2020). In FIESC, although the morphotype of *F. Incarnatum* and *F. equiseti* has been documented (Avila et al., 2019), it is still difficult to differentiate between some ambiguous species (Jiang et al., 2020).

Fusarium isolates that produce slightly slender, relatively straight-to-curved macroconidia comprising five septations and curved and foot-shaped cells i.e., apical and basal cells, respectively, are categorized as FSSC. In this species complex, *F. asiaticum* produces straight to slightly curved macroconidia with five septations comprising slightly curved apical cells and well-developed basal cells (Li et al., 2017). The morphology of *F. asiaticum* is very similar to *F. graminearum*, and they exhibit slightly dissimilar conidial features.

## 4. Molecular characterization

Advanced methods must be devised and applied for rapid plant disease diagnosis to reduce crop loss brought on by pathogen infection (Shakeel et al., 2022a). The characterization of *Fusarium* species that are associated with bakanae rice disease purely based on morphological features is a difficult task because of the high diversity in their characteristics (Choi et al., 2018). Consequently, the systemic culture media and standard methodology, in addition to multi-locus molecular records, are currently considered imperative for assertive species-level identification. Even though several studies have established that the species in FFSC associated with bakanae disease of rice, along with several other *Fusarium* species (*F. moniliforme, F*. *thapsinum, F. commune*, and *F. pseudonygamai*), can be differentiated using just the gene sequence of the *TEF1* gene (Wulff et al., 2010; Choi et al., 2018), but there is no evidence that the *TEF1* gene can be used to characterize all species in Fusarium species complex (FSC). Nevertheless, a combination of the *RPB1* and *RPB2* genes was employed to deduce the phylogenetic relationships of the species in FSC (O'Donnell et al., 2013), and an amalgamation of the *RPB1, RPB2*, and *TEF1* genes were utilized to analyze the genetic diversity of *Fusarium oxysporum* f. sp. *Cubense*; this method, therefore, has been highly endorsed (Maryani et al., 2019). Furthermore, with a lack of reference sequences available for the RPB1 gene in *F. incarnatum*, six species that were identified based on their morphological characters and by the *TEF1* gene were first categorized into the FSC through the use of just *RPB1* and *RPB2* genes because of the lack of individual reference sequences for the species. Further analysis of each FSC was further done *via* several loci sequences (Jiang et al., 2020).

## 5. Virulence variation

In various studies, the pathogenicity of several *Fusarium* species (*F. fujikuroi, F. proliferatum, F. equiseti, F. incarnatum, F. commune, F. andiyazi*, and *F. asiaticum*) was assessed to determine the virulence discrepancies among species. After 20 days of germination, rice seedlings inoculated with *F. fujikuroi* showed typical symptoms of bakanae disease (Choi et al., 2018; Jiang et al., 2020), while *F. andiyazi* and *F. proliferatum* inoculation caused the specific yellowing of leaves along with a few dying leaves to appear on normal-height rice seedlings. Comparative stunted plants with yellowish leaves were reported in response to the inoculation of *F. incarnatum* and *F. asiaticum* on seedlings. These findings from several studies showed that these six species were pathogens of rice and capable of causing the disease, but their virulence varied (Jiang et al., 2020). *F. fujikuroi* as a cause of bakanae disease of rice along with other associated species has been studied by many researchers. Numerous studies have revealed that other species of FSC (including *F. verticillioides, F. proliferatum, F. andiyazi, F. equiseti, F, incarnatum*, and *F. asiaticum*) were concomitant with the rice bakanae disease (Prà et al., 2010; Wulff et al., 2010; Choi et al., 2018; Jiang et al., 2020). These associated species could develop characteristic symptoms of bakanae but their symptoms varied significantly. Additionally*, F. fujikuroi* stimulated elongation in the seedlings, whereas *F. proliferatum* and *F. andiyazi* isolates were found to cause the comparative stunting of rice seedlings (Choi et al., 2018; Jiang et al., 2020). Significant stunting was observed in seedlings inoculated with *F. asiaticum, F. equiseti*, and *F. incarnatum*. Moreover, *F. concentricum* also can cause characteristic symptoms of bakanae disease in rice (Jeon et al., 2013), and *F. incarnatum* has also been found as a pathogen of bakanae (Song et al., 2014). *F. equiseti* is termed a saprophytic microbe (Choi et al., 2018), whereas the saprophytic or pathogenic role of *F. asiaticum* has not been recognized. The cause of virulence variation among species was determined through germination tests under *in vitro* conditions. *F. asiaticum* (73%) and *F. andiyazi* (71%) showed the maximum inhibition percentage of seed germination followed by *F. incarnatum* (54%), *F. equiseti* (44%), and *F. proliferatum* (35%), all of which also showed significant inhibition, while *F. fujikuroi* showed the least inhibition of seed germination (Zhang et al., 2015). Meanwhile, no significant difference was found between the inhibition percentages of seed germination by *F. asiaticum* and *F. andiyazi*. The cause of the inhibited seed germination mechanism was found to be associated with mycotoxins produced by the particular species (Jiang et al., 2020).

## 6. Mycotoxin production

A variety of mycotoxins produced by the *Fusarium* species have been associated with the bakanae disease. Within the FFSC, *F. fujikuroi* is the only species that produces Gibberellin A3, which encourages plant elongation. Among several mycotoxins [such as fumonisins (FBs), enniatins (ENN) beauvericin (BEA), and moniliformin (MON)], at least one mycotoxin has been produced by FFSC isolates (Choi et al., 2018; Saito et al., 2021). Although fusaric acid, moniliformin, and fumonisins were found to cause phytotoxicity in plants and develop characteristic symptoms (Saito et al., 2021), no information existed regarding their effect on the germination of rice seeds. Isolates of FIESC from rice seeds can produce trichothecenes, such as 4-acetylnivalenol (4-ANIV), nivalenol (NIV), 15-acetyldeoxynivalenol (15-ADON), 3-acetyldeoxynivalenol (3-ADON), and deoxynivalenol (DON) (Avila et al., 2019). In another study, the production of T-2 toxin by *F. equiseti* was evident but not DON. Nonetheless, most of the isolates of *F. asiaticum* (member of FSSC) isolated from Jiangsu, China have been found to primarily produce 3-acetyldeoxynivalenol (3-ADON), while others produced nivalenol (NIV) (Dong et al., 2020). Various trichothecenes have been recognized as phytotoxic, while some others such as 4,15-Diacetoxyscirpenol (4,15-DAS) have been demonstrated to impede soybean seed germination (Miedaner et al., 2017; Jiang et al., 2020).

## 7. Disease mechanism

To prevent host plants from responding to infection, Fusarium spp. has developed sophisticated strategies. The pathogens use a wide variety of infection tactics to successfully infiltrate and colonize the host. Aside from the core genome, which is involved in fundamental metabolism, the Fusarium genome also contains virulence-related areas (adaptive genome) (Leslie and Summerell, 2013; Beccari et al., 2022). Effectors play a vital role in the interactions between the plant and the pathogenic fungus. The study of fungal plant diseases has resulted in several effectors being discovered, some of which have been described for their virulence roles (Tariqjaveed et al., 2021; Shakeel et al., 2022b). Fusarium has numerous endophytic and positive interactions with host plants, but there have been few molecular-level studies of these interactions (Pereira et al., 2019). Characterizing the secretome aids in the comprehension of pathogen virulence and host plant infection mechanisms. For instance, it has been anticipated that a subset of pathogen-secreted proteins can determine the success and progress of disease development. The size of the anticipated secretome (*F. graminearum*) is 574 proteins, or 4.2% of its total anticipated gene repertoire (Brown et al., 2012; Bashyal et al., 2017). The total anticipated secretory proteins are 1,336 in the genome of IMI58289 *F. fujikuroi* isolate (Wiemann et al., 2013; Bashyal et al., 2017). However, the investigation into the role of secretome (i.e., plant cell wall degrading enzymes) in *F. fujikuroi* has been confined to the detection of genes number. It has been indicated that, in *F. fujikuroi*, the secretome is composed of distinct proteins that interact in an organized way to inhibit various characteristics of plant immunity to effectively cause the disease. In addition, the results suggest that most of these genes are activated during the host-pathogen interaction when the genome appears to be enriched with cell envelope (CE) enzymes and glycoside hydrolases (GHs) that can penetrate the cell barrier of the host plant. The GH3 and GH5 abundance stimulate the disruption of pectin, cellulose, and hemicellulose, suggesting that these degrading enzymes play crucial roles in the genome of *F. fujikuroi* (Bashyal et al., 2017). Additionally, *F. fujikuroi* possesses one each of the GH67 and GH36 genes encoding α-galactosidases that are lacking in the majority of plant pathogens. The presence of abundant families of pectin degrading enzyme GH78 (1 gene), PL9 (2 genes), CE8 (4 genes), PL3 (5 genes), GH28 (8 genes), and PL1 (10 genes) may aid *F. fujikuroi* in root tissue colonization. The existence of CAZymes families such as GH10 (1 gene), GH36 (1 gene), GH53 (1 gene), GH62 (1 gene), GH7 (2 genes), GH12 (3 genes), PL3 (5 genes), PL1 (10 genes), and CE5 (12 genes) suggests that *F. fujikuroi* possesses an abundance of cutinases and plant biomass-degrading enzymes (Bashyal et al., 2017).

After 7–10 days of inoculation, the secretomes, PL (genes 5526, 3064), CBM (genes 13528, 12667, 11810, 5178), GH (gene 12698), CE (gene 12149), and gene 9849, associated with increased virulence were expressed maximally (Bashyal et al., 2017). This increase was stable with the richness of pectins in cell walls and could be mainly ascribed to the enhancement of pectin-degrading enzymes. This expression pattern also recommended that particular enzymes may have distinct roles at diverse infection stages. The higher stimulation of genes of secretomes in the susceptible rice genotype than the resistant genotype during the early stage of inoculation may be a result of greater colonization of the pathogen in the susceptible rice genotype and upregulation of the secretomes (Bashyal et al., 2017). Some genes, such as 12,587 (GH-72 family) and 1,530 (AA-7 family), have a greater expression in resistant genotypes than in susceptible genotypes. Maximum gene expression was observed 30 days after inoculation. It was found that the local and systemic behaviors of *F. fujikuroi* was associated with the genotype of rice during the pathogen-host interactions, which may be further involved in altering the expressed genes number and their level of expression (Matic et al., 2016a). Moreover, it was reported that the profiles of gene expression were different at different times of the infection, and it appeared that the expression of particular genes that encoded the degrading enzymes gradually deteriorated the host plant during the infection (Bashyal et al., 2017).

## 8. Control methods

### 8.1. Host resistance

The most eco-friendly and cost-efficient control method is the use of a resistant host plant. In Pakistan, *in vivo* screenings were conducted for the detection of Pakistan rice varieties having resistance against bakanae disease, which showed that DR-82, DR-83, DM-15-1-95, IR-6, IR-8, KS-133, and KS-282 were the resistant rice varieties (Iqbal et al., 2011; Lee et al., 2022). In Korea, the conidia of *F. fujikuroi* were inoculated in different rice varieties through the tissue embedding method to evaluate the resistance, and it was found that Wonseadaesoo, Erguailai, and Gwangmyeongbyeo have resistance (Hur et al., 2016). In another study, through comparative transcriptome analyses, Dorella was found susceptible while Slelnio was the resistant rice cultivar against bakanae (Matic et al., 2017). Plants activate different defense systems in response to pathogen invasion, which can lead to an increased resistance host phenotype, particularly in genotypes with resistant loci (Lee et al., 2022). In resistant varieties, the host plants resist the bakanae disease of rice by activating their defense system.

**Figure 3:**
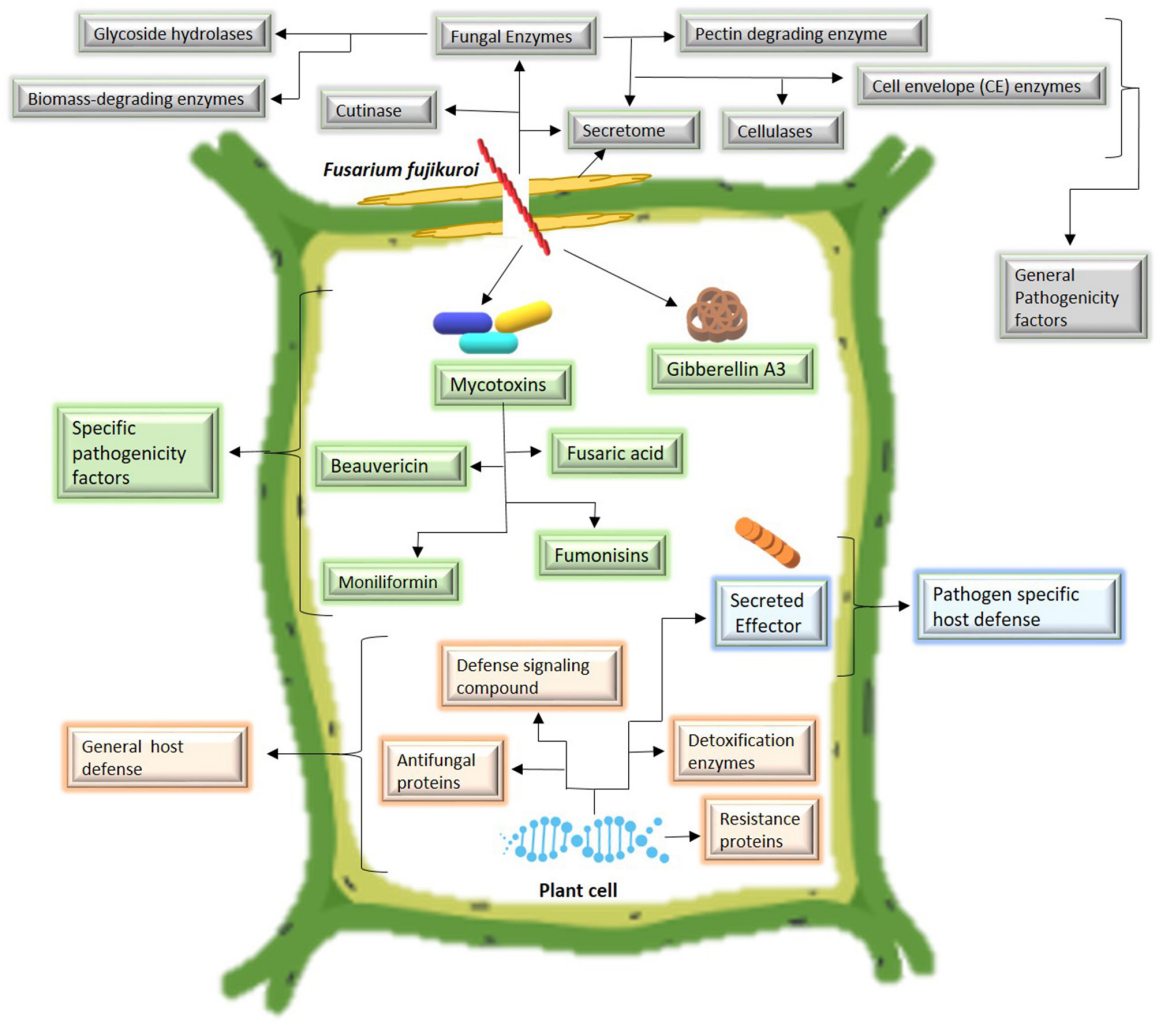
Mechanism of pathogen colonization and plant host defense.

To invade the host, Fusarium species secrete cell wall degrading enzymes or secretomes including fungal enzymes (pectin degrading enzymes, cellulase, glycoside hydrolysis, biomass-degrading enzymes, cell envelop enzymes, etc.) and cutinase to disrupt the rigid plant cell wall. All these compounds are categorized as general pathogenicity factors because several Fusarium species produce such compounds to facilitate host invasion (Chang et al., 2018). After host invasion, some specific metabolites are produced by particular Fusarium species. The most important secondary metabolites produced by *F. fujikuroi* include mycotoxins, such as beauvericin, fusaric acid (Niehaus et al., 2017), and fumonisins (Suga et al., 2019), and plant hormones such as gibberellins A3 (Suga et al., 2019). Mycotoxins produced by other FFSCs include fumonisins by *Fusarium proliferatum* and *F. verticillioides* (Katoch et al., 2019). It is still unknown what function fumonisins play in the life cycle of fungi or their pathogenicity. For instance, *F. andiyazi* produces no fumonisins or gibberellins (Masratul Hawa et al., 2013). Mycotoxin production by *F. commune* is still unclear (Niehaus et al., 2017). To endure the pathogen attack, resistant cultivars exhibit two types of defense systems: First, a general host defense in which several antifungal compounds including resistance proteins, antifungal proteins, defense signaling compounds, and detoxification enzymes are produced by the host in response to pathogen attack. These compounds are not specific for a particular *Fusarium* species and are effective against various Fusarium species; second, a pathogen-specific host defense in which host plants secrete specific effector proteins for a particular *Fusarium* species. Such defense mechanisms enable the host to withstand the infection. The pathogen colonization and the host-defense mechanism are shown in Figure 3.

### 8.2. Quantitative trait loci

Vertical resistance in rice varieties conferred by a single resistant gene (R-gene) has been reported to be gradually overwhelmed by new or resistant pathological races (Lee et al., 2018, 2019). Therefore, it is necessary to understand the resistance mechanism by identifying the resistance genes which also aids in assessing the marker efficacy used in rice breeding. Several studies have reported the resistance conferred by quantitative trait loci (QTL) against rice bakanae disease. Based on genetic mapping methods, population types, and marker systems, *qFfR1* and *qB1* (Ji et al., 2018), *qBK1* (Hur et al., 2015; Lee et al., 2019), *qBK1*^*z*^ (Lee et al., 2021), *qBK1*^*WD*^ (Lee et al., 2018), *qBK1_628091* (Volante et al., 2017), *qBK1.1, qBK1.2*, and *qBK1.3* (Fiyaz et al., 2016) on chromosome 1, *qB4* on chromosome 4 (Volante et al., 2017), *qFfR9* on chromosome 9 (Kang et al., 2019), and *qB10* on chromosome 10 (Chang-deng et al., 2006) have been identified as QTL associated with rice resistance against bakanae disease. Since *qBK1* and *qBK1.1* were found in the same genomic region, they might be the same QTL (Fiyaz et al., 2016). Recently, *qBK4*^*T*^ was detected on chromosome 4 as a novel locus by genetic mapping and the Genome-Wide Association Study approach. Additionally, *qBK4.1* (Grant, 2006) and *qBK4_31750955* (Volante et al., 2017) have also been detected in chromosome 4 but their position differs from *qBK4*^*T*^ (Lee et al., 2022).

### 8.3. Bio-control

**Figure 4:**
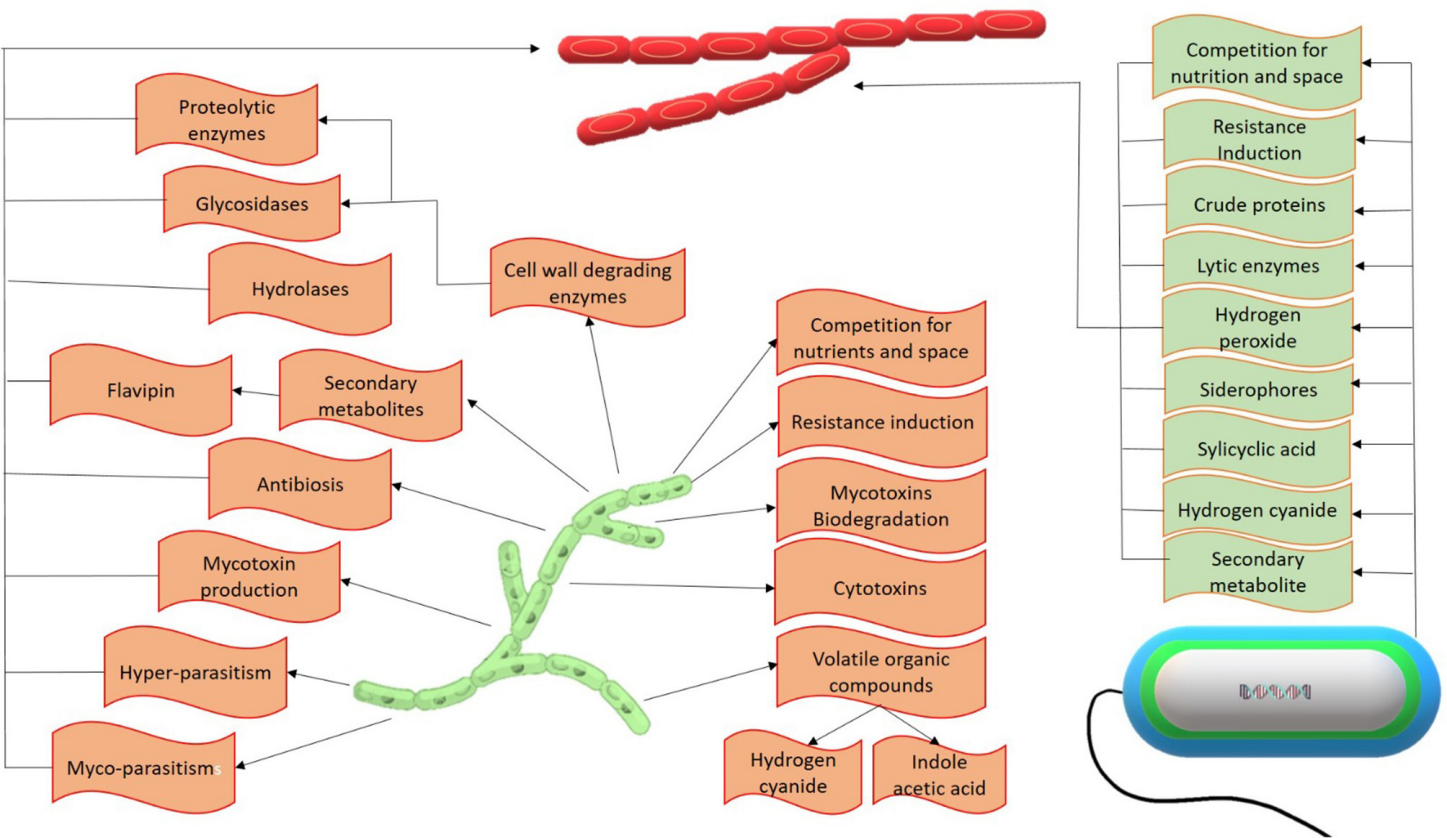
Antifungal mechanism of biocontrol agents against *F. fujikuroi*.

Biocontrol is one of the most eco-friendly, convenient, and effective control methods for the bakanae disease of rice. Various bacterial and fungal biocontrol agents have been found to have higher antifungal efficacy and significantly inhibited *F. fujikuroi*. The general antifungal mechanism of biocontrol agents involved in the *F. fujikuroi* suppression can be observed in Figure 4.

#### 8.3.1. Bacterial bio-control agents

Several bacteria have been reported to demonstrate an antagonistic effect against the bakanae disease of rice (Table 1). In a study, strains of *Pseudomonas fluorescens* and *Pseudomonas putida* were found to effectively suppress *F. fujikuroi* growth (Safari Motlagh and Dashti, 2020). It was further revealed that *Paenibacillus polymyxa*, which is an endophytic bacterium, also has the efficacy to reduce the growth of *F. moniliforme* (Zhang et al., 2010). Several Bacillus species including *B. subtilis, B. circulans* (Safari Motlagh and Dashti, 2020), *B. megaterium* (Luo et al., 2005), *B. oryzicola* (Hossain et al., 2016), and *B. cereus* (Etesami and Alikhani, 2017) were observed to successfully inhibit the *F. fujikuroi* growth (Nawaz et al., 2022). In another study, it was found that the B-44 strain of *B. subtilis* has the potential to effectively reduce the disease incidence of rice bakanae under greenhouse conditions. Moreover, the QST 713 strain of *B. subtilis* has been commercially applied as a bio-fungicide i.e., in the context of Serenade against bakanae disease of rice (Matić et al., 2014). Studies have further reported that the application of the YC7007 strain *B. oryzicola* in nursery boxes and pots to control bakanae disease decreased the disease severity from 46% to 78% (Hossain et al., 2016).

**Table 1:** Bacterial bio-control agents used against the bakanae disease of rice.

	**Species**	**Conditions**	**Application method**	**Disease inhibiton**	**Antagonistic mechanism**	**References**
Bacterial bio-control agents	*Bacillus circulans*	Greenhouse	Seed treatment	–	–	Safari Motlagh and Dashti, 2020
	*Bacillus cereas*	*In vitro*	Dual culture	66%	–	Etesami and Alikhani, 2017
	*B. oryzicola*	*In vivo*	Root drenching	46-78%	Hydrogen peroxide secretion, Systemic resistance induction	Hossain et al., 2016
	*Bacillus megaterium*	*In vitro*	–	–	–	Luo et al., 2005
	*Bacillus subtilis*	*In vitro*	Dual culture	76%	Stimulation peroxidase activity, production of lysis enzymes	Sarwar et al., 2018, Safari Motlagh and Dashti, 2020
	*P. fluorescens*	*In vitro*	Dual culture volatile antibiotics	69%	Production of volatile compounds (hydrogen cyanide), sidrophores, salicylic acid, lytic enzyme	Safari Motlagh and Dashti, 2020
			Diffusible antibiotics	77–78%		
	*P. putida*	*In vitro*	Dual culture	59.21%	Production of antibiotics	Safari Motlagh and Dashti, 2020
	*P. polymyxa*	*In vitro*	Dual culture	–	Production of crude protein	Zhang et al., 2010

#### 8.3.2. Fungal bio-control agents

Previously studied fungi as bio-control agents to control bakanae disease of rice are presented in Table 2. Trichoderma is a widely studied fungal bio-control agent to control several diseases. The antagonistic potential of the *Trichoderma* species against phytopathogenic fungus (*F*. *fujikuroi*) was evaluated by applying the SKT- 1 strain of *T. asperellum* in the form of commercialized Eco-hope, and it was found that this bio-control agent disrupted the pathogen cell wall after penetrating the pathogen hyphae (Win et al., 2021). Application of *T. virens* (Safari Motlagh and Roshani, 2020) and *T. harzianum* (Wan et al., 2015) individually suppressed the bakanae disease of rice; however, their antagonistic efficiencies were increased by applying them in combination with the chemical thiophanate-methyl 80% WP at 2 g/L. According to Ng et al. (2015), the investigation and application of all selected strains of *Trichoderma* sp. significantly suppressed the bakanae disease. Yeasts including *Pichia guilliermondii, Metschnikowia pulcherrima*, and *Sporidiobolus pararoseus* also show antagonistic potential against various pathogens such as *F*. *fujikuroi* (Zhang et al., 2011; Matić et al., 2014). Application of the R9 strain of *P. guilliermondii* and R23 strain of *M. pulcherrima* in combination with thermotherapy of rice seeds for 10 min at 60°C resulted in additionally decreasing the disease incidence of bakanae to 5% than applying these bio-control agents alone (Matić et al., 2014). *Stachybotrys atra, Penicillium thomii*, and *Penicillium chrysogenum* were found to have antagonistic efficacy against *F. moniliforme*. Two strains of *Chaetomium globosum*, including NR-R688 and NR-SH321, strain NR-L645 of *Fusarium* sp., and strain NR-L243 of *Penicillium* sp., decreased the incidence of rice bakanae to ~2–6% (Ramesh et al., 2020). Furthermore, strain W5 of *Fusarium commune* was found to be non-pathogenic with the efficacy to control bakanae disease. For instance, the application of strain W5 by spraying on rice flowers and seedlings inhibited the hyphal growth of *F. fujikuroi*, and the antagonist could survive in rice seeds for ~6 months or more (Saito et al., 2021).

**Table 2:** Fungal bio-control agents used against the bakanae disease of rice.

	**Species**	**Conditions**	**Application method**	**Disease inhibiton**	**Antagonistic mechanism**	**References**
Fungal bio-control agents	*C. globosum*	*In vitro*	Dual culture	97.4%	Secretion of secondary metabolite flavipin	Ye et al., 2013; Ramesh et al., 2020
	*S. pararoseus*	Greenhouse	Seed dressing	40.5%	Organic volatile compounds production, competition	Huang et al., 2012; Matić et al., 2014
	*P. guilliermondii*	Greenhouse	Seed dressing	73%	Competition for nutrients and space, Biodegradation of mycotoxins, secretion of cell-wall degrading enzymes	Matić et al., 2014; Matic et al., 2016b
	*M. pulcherrima*	Greenhouse	Seed dressing	64.5%	Production of hydrolases (cell wall degrading enzymes), competition,	Matić et al., 2014; Matic et al., 2016b
	*Penicillium* sp. (strain NR-L243)	*In vitro*	Dual culture	92.3%	Production of mycotoxins	Ramesh et al., 2020
	*P. chrysogenum*	*In vitro*	Dual culture	–	Penecillin production	Mangiarolti et al., 1987
	*P. thomii*	*In vitro*	Dual culture	–	–	Mangiarolti et al., 1987
	*Trichoderma virens*	Greenhouse	Seed dressing	79%	Hyperparasitism, production of volatile compounds, IAA, hydrogen cyanide	Ng et al., 2015; Safari Motlagh and Roshani, 2020
	*T. asperellum*	*In vitro*	Dual culture	–	Mycoparasitism, Degradation of the cell wall by glycosidases	Yang and Xu, 2013; Win et al., 2021
		*In situ*				
	*T. harzianum*	Greenhouse	Seed dressing	83%	Hyperparasitism, antibiotic production, hydrolytic enzymes secretion	Ng et al., 2015; Wan et al., 2015; Safari Motlagh and Roshani, 2020
		In Vitro	Dual culture	>60%		
	*Trichoderma viride*	*In vitro*	Dual culture	–	Production of lipase, proteolytic enzymes	Rajathi et al., 2020; Safari Motlagh and Roshani, 2020
	*Talaromyces* sp. isolate KNB-422	*In vivo*	Seed Treatment	–	Mycoparasitism	Kato et al., 2012
	*T. flavus*	Glasshouse	Seed Treatment	70%	Volatile metabolite production	Naraghi et al., 2013; Rawat et al., 2022
	*S. atra*	*In vitro*	Dual culture	–	Production of cytotoxins e.g., trichothecenes	Mangiarolti et al., 1987; Jarvis, 1991
	*Fusarium commune* (strains W3, W5)	*In vivo*	Aerial Spray	–	Resistance induction	Saito et al., 2021
	*Fusarium* sp. (strain NR-L645)	*In vitro*	Dual culture	92.3%	–	Ramesh et al., 2020

### 8.4. Divergent invasion patterns of *F. fujikuroi* (CF283-GFP) in the early infection

Seeds of the susceptible and resistant cultivars were injected with the fungus pathogen CF283 isolate tagged with a green fluorescent protein to assess the invasion of the *F. fujikuroi* (GFP). Confocal imaging of rice seed embryo sections showed that the fungal pathogen entered both the Ilpum and Tung Ting Wan Hien1 embryos shortly after inoculation. At 3 and 7 dpis of Ilpum, the coleoptile showed robust pathogen colonization localized by the green fluorescence of the GFP signal. In contrast, under identical circumstances, the resistant Tung Ting Wan Hien1 showed no clear signs of colonization in the coleoptile. The intricate process of adhesion, penetration, and subsequent colonization inside cells and in intercellular compartments is necessary for the infection of *Fusarium* spp. (Jansen et al., 2005; Rana et al., 2017; Lee et al., 2022). According to research conducted by Lee et al. (2018), vulnerable rice cultivars have more *F. fujikuroi* in their stems than resistant ones. Similarly, the aerenchyma, pith, cortex, and vascular bundle of the rice sheath and stem were also found to be the best places for *F. fujikuroi* to thrive. In the first phase of infection, the spread of *F. fujikuroi* in seeds from susceptible and resistant cultivars was assessed. The study by Lee et al. (2022) found a comparable amount of the target fungal infection in the embryo based on the cellular localization of *F. fujikuroi* strain CF283 tagged with a GFP. Later on, it was discovered that the pathogen quickly colonized the coleoptile of Ilpum at 3 and 7 days after infection, but in the resistant cultivar Tung Ting Wan Hien1, the presence of GFP-*F. fujikuroi* in the coleoptile under the same circumstances was incredibly poor. In earlier investigations (Elshafey et al., 2018; Lee et al., 2018), *F. fujikuroi* was shown to have a similar pattern of colonization, with its presence being viewed in the vascular bundles, mesophyll, and subcutaneous tissue of infected stems in susceptible cultivars as opposed to the resistant one. This led to the hypothesis that soon after inoculating rice seeds with the virulent isolate CF283 of *F. fujikuroi* in both resistant and susceptible varieties, the pathogen colonized quickly in susceptible varieties (Ilpum), which had a defective or weak defense system, but the observed resistance of Tung Ting Wan Hien1 could be partially explained by the contribution of qBK4T.

### 8.5. Defense mechanism

Research on the bakanae disease highlights some of the defensive mechanisms used by the resistant cultivar C101A51 in response to *F. fujikuroi* infection. In comparison to the resistant genotype C101A51, a substantial number of peroxidase genes were found in the vulnerable genotype Rasi. Since peroxidases are needed to stop pathogen diffusion within cells, the limited pathogen spread in resistant genotypes may be the reason behind it. Similarly, Rasi and C101A51 both showed higher numbers of genes associated with chloroplasts. By reducing the supply of accessible carbon for pathogen development and redirecting the route toward defensive responses, it may be possible to consider a decrease in the expression of chloroplastic genes as a resistance strategy of the rice plant (Bolton, 2009; Bashyal et al., 2022). The resistant genotype showed higher levels of heat stress-related transcripts (BIP4-like protein, transcription factor A-2a-like, and BHLH148) than the susceptible genotype. Transcriptional regulators of heat stress have also been linked to resistance to fungi in many plant species. For instance, the bakanae resistant genotype Selenio has been discovered to have upregulated transcription factors associated with heat stress (Matic et al., 2016a; Bashyal et al., 2022).

Antimicrobial peptides were also expressed greater in C101A51 and were encoded by gene ID BGIOSGA033746. Plants generate antimicrobial peptides, also known as host defense peptides, as a first line of protection against possible dangerous bacteria. Several genes were upregulated in the resistant genotype C101A51, including cysteine proteinase inhibitor 10 (LOC4335551), disease resistance protein TAO1-like (LOC112939055), oleosin 16 kDa-like (LOC4336570), pathogenesis-related protein (PR1), pathogenesis-related protein (PR4), and BTB/POZ and MATH domain-containing protein 5-like (LOC112936838). One explanation for their admission might be due to the decline in their expressiveness. CTP is a particular inhibitor of cysteine proteinases that plays a part in the control of endogenous processes as well as the defense against infections and pests. RGA3, a potential disease-resistant protein similar to TAO, was expressed more often in the resistant genotype C101A51 (Bashyal et al., 2022). In the Pseudomonas syringe-rice interaction, it was found that TAO was responsible for the expression of the pathogenesis-related protein 1 (PR1) gene. This finding is suggestive of TAO's role against *F. fujikuroi* in rice, as more expression of the oleosin-16 kDa-like protein in resistant genotypes is suggested, and more oil bodies are present in the seeds of resistant rice genotype C101A51. Compared to control plants, infected plants showed higher amylase activity. The production of storage starch granules during rice seed maturation and the motivation of the stored starch to nourish the sprouting seedlings during seed germination were both influenced by amylase isozymes, which have a direct impact on plant development and yield. Gibberellic acid content often affects amylase activity (Bashyal et al., 2022). The enzymatic activity may have been boosted by the *F. fujikuroi*-infected plant's elevated gibberellic acid concentration. Microtubule-related proteins called cytoplasmic linker associated proteins (CLASPs) are crucial for controlling the dynamics of microtubules, which are crucial for plant growth and development. Shorter internodes and semi-dwarfism in plants were linked to the inhibition of CLASP protein, according to Zhu et al. (2018). The elongated plant phenotype in rice genotype Rasi could have been caused by CLASP transcript expressions that were noticeably positive. Proteins with the BTB/POZ and MATH domains were more abundant in the resistant genotype C101A51. Broadly speaking, the BTB domain is involved in how plants react to biotic and abiotic stressors. This gene upregulation in resistant genotypes may aid in their defense against *F. fujikuroi* (Bashyal et al., 2022).

### 8.6. Prediction and analysis of *F. fujikuroi* secretome

SignalP version 4.1 was able to classify 1,207 proteins out of the 13,603 proteins as conventional secretory proteins, whereas TargetP version 1.1 identified 2,265 proteins as secretory proteins. We scanned 2,265 proteins using TMHMM software after the filtered sets (SignalP and TargetP) were combined and duplicate segments were eliminated. Once 574 transmembrane proteins were excluded from the protein data set, a total of 1,691 sequences were predicted to be secretory proteins. Using WoLFPSORT version 3, secretory proteins identified in the preceding phase were further screened, yielding 1,194 proteins. Approximately 985 potential secretory proteins were given GO keywords in three GO categories, including molecular function (736), biological process (670), and cellular component for the analysis of the anticipated *F. fujikuroi* secretome (280). Single-organism processes, cellular metabolic processes, cellular processes, biological regulation, regulation of biological processes, cellular component organization or biogenesis, response to stimulus, and localization were among the categories with the greatest representation under the biological process (Bashyal et al., 2017).

**Figure 5:**
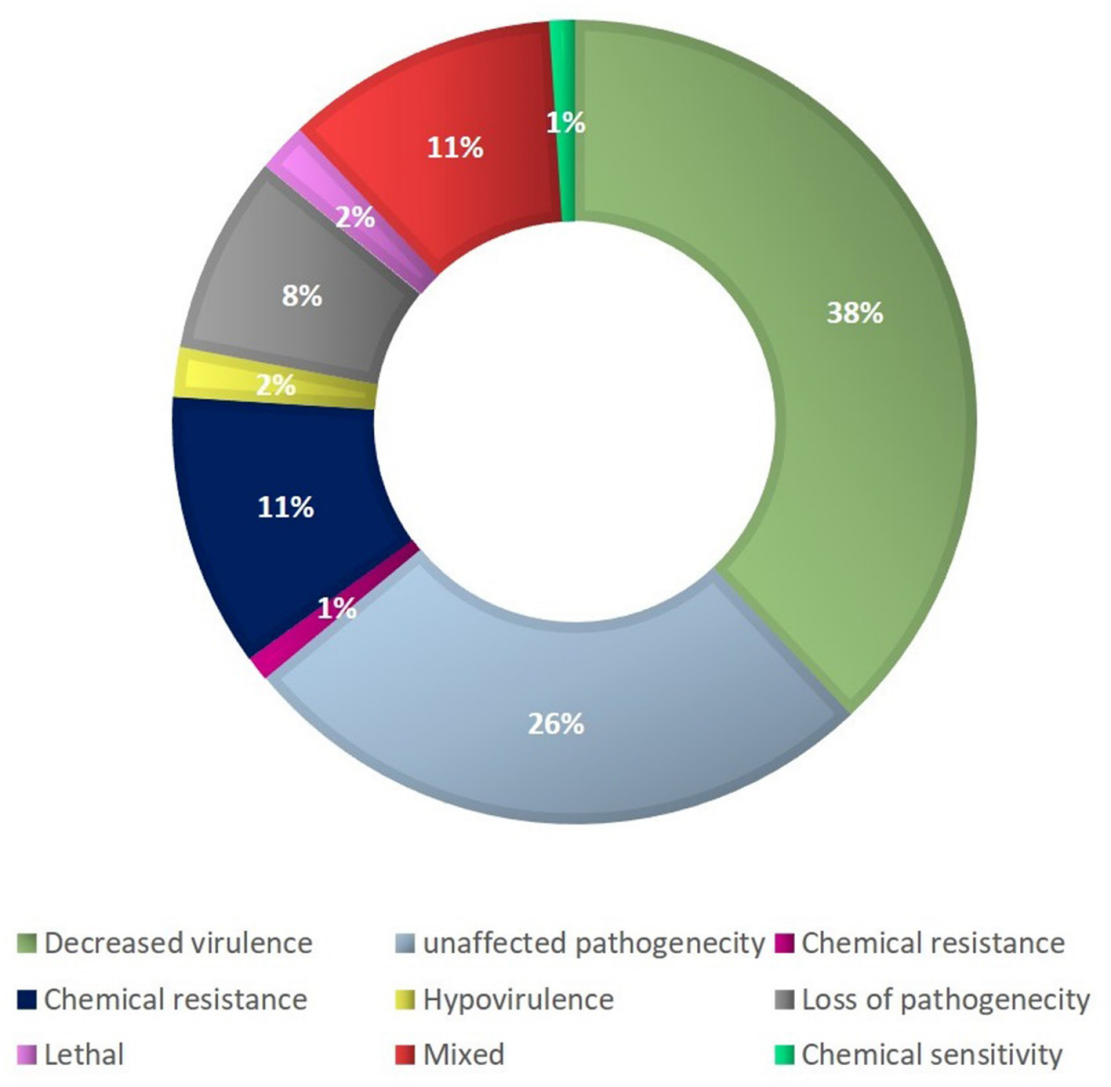
Categories of secretome genes of *F. fujikuroi* (Bashyal et al., 2017).

The most prevalent proteins in the molecular function ontology were those involved in binding, antioxidant activity, transporter activity, and structural molecule activity. Proteins for the membrane part, cell and cell part, organelle part, and macromolecular complex were significantly abundant in the cellular component category. Approximately 585 proteins out of 1,194 secretory proteins had demonstrated matches with PHI-d at a base in various categories. Of them, 38% of the proteins were associated with decreased virulence, 26% with unaltered pathogenicity, and 11% with proteins of mixed character. The presence of 5% polysaccharide lyases (PLs), 7% glycosyl transferases (GTs), 16% auxiliary activities (AAs), 11% carbohydrate-binding modules (CBMs), 20% carbohydrate esterases (CEs), and 41% glycosyl hydrolases (GHs) were predicted in the *F. fujikuroi* “F250” secretome (Figure 5) using the CAZy database and HMMER scan based on the profile compound for the six CAZy classes. Further investigations were done for the families of enzymes that break down cell walls. We found 16 of the 51 GH families to exist, which exhibited the presence of three or more genes. The GH16 family had the most genes (15), followed by GH43 (14 genes), GH5 (11 genes), and GH3 (10 genes) (9 genes) (Figure 5).

The secretome of *F. fujikuroi* contained members of 10 CE families. The most genes (i.e., 18) were found in CE10, which was followed by CE1 (16 genes), CE5 (12 genes), and CE16 (6 genes). Moreover, four PL families were predicted: PL1 (10 genes), PL3 (5 genes), PL4 (2 genes), and PL9 (2 genes). In addition, classes including AA (7 families), CBM (16 families), and GT (25 families) that play indirect roles in the degradation of carbohydrates were found (Bashyal et al., 2017). The CBM-01, AA7, and GT-34 families were the most prevalent of these. There were many oxidoreductases, transferases, hydrolases, and lyases in the secretome of F. *fujikuroi* as well. Accordingly, the research conducted by Bashyal et al. (2017) revealed that the secretome of *F. fujikuroi* contained a variety of proteins that may aid in the fungus' appropriate colonization, the breakdown of host plant materials to get nutrients, and the inactivation of the host's defenses. Following several species of *F. oxysporum*, the secretome of the *F. fujikuroi* isolate “F250” is closely connected to that of the *F. fujikuroi* isolate IMI58289.

### 8.7. Chemical control

#### 8.7.1. Benzimidazoles

**Figure 6:**
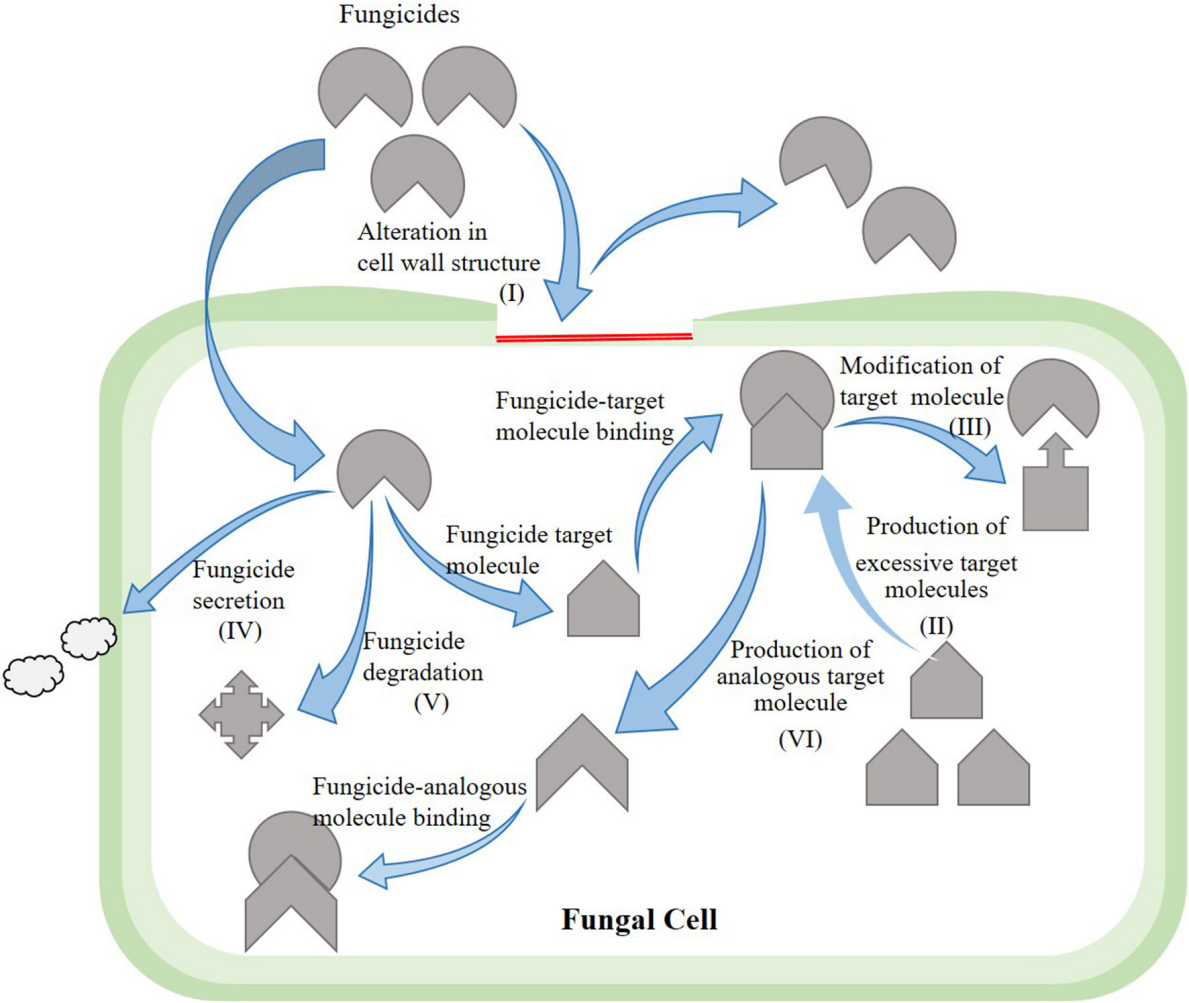
Mechanism of resistance development in fungi against fungicides.

Currently, control of rice bakanae disease through a chemical application is the most common control method. The application of broad-spectrum fungicides (benzimidazoles) against the bakanae disease of rice has been under use for decades (Saito et al., 2021). Benzimidazoles interrupt meiosis and mitosis in pathogen cells and damage cellular processes, including cell division, formation of the cytoskeleton, and intracellular transfer. The typical benzimidazole fungicides are carbendazim, benomyl, fuberidazole, thiabendazole, and thiophanate-methyl. Under *in vitro* conditions, the application of fungicides suspension at 0.3%, such as thiophanate-methyl at 80% and carbendazim at 50%, as a seed treatment suppressed the bakanae disease (Latif et al., 2021); however, seed dipping treatment for 10 min in 10 g/L benomyl completely suppressed the bakanae infection. Seed treatment with the chemical thiabendazole was also effective against the bakanae disease (Iqbal et al., 2013). However, due to excessive application of benzimidazoles fungicide, *F. fujikuroi* strains resistant to such fungicides have been reported. Potential mechanisms of resistance in phytopathogenic fungi to fungicides include measures such as (a) alteration in the structure of fungal cell wall due to which fungicide cannot enter the fungal cell wall, (b) production of excessive target molecule due to which excessive production of fungicide become unable to completely suppress the activity of target molecule, (c) modification of the target molecule due to which altered structure fungicide cannot bind itself with the target molecule, (d) secretion of fungicide whereby efflux pumps excrete the fungicide out of the fungal cell, (e) degradation of fungicide due to which fungicide loses the potential to perform its activity, and (f) production of an analogous target molecule in fungal cells due to which the fungicide cannot identify its target molecule and binds with the alternative molecule (Figure 6). In *F*. *moniliforme*, benomyl resistance is reported to be conferred by β-tubulin mutation D50Y (Yan and Dickman, 1996). Similarly, Chen et al. (2014) revealed that mutations F200Y and E198V in β2-tubulin produced resistance to carbendazim in Chinese *F. fujikuroi* (Table 3).

**Table 3:** Fungicides used against the bakanae disease of rice.

**Fungicide group**	**Chemical compound**	**Mode of action**	**Resistance mechanism**	**References**
Benzimidazole	Benomyl (benlate)	Inhibit microtubule formation, apoptosis induction	D50Y mutation in β-tubulin	Yan and Dickman, 1996; Kara et al., 2020
	Thiabendazole (tresaderm, mintezol, arbotect)	Inhibit mitochondria activity, inhibit protein synthesis	–	Iqbal et al., 2013
	Carbendazim (bavistin, derosal, haydazin, knowin)	Inhibit microtubule formation	F200Y and E198V mutations in β2-tubulin	Chen et al., 2014; Latif et al., 2021
	Thiophanate-methyl (Topsin M)	Degradation of β-tubulin, interferance in Glycolysis	–	Iqbal et al., 2013; Latif et al., 2021
Others	Tebuconazole (Folicur)	Interferes in biosynthesis of ergosterol	–	Hossain K. S. et al., 2015
	Chitosan oligosaccharides	Induction of callose deposition that stimulates plant immunity	–	Luna et al., 2011; Kim et al., 2016
	Metiram (Arbatene)	–	–	Hossain M. S. et al., 2015
	Ethylenediaminetetraacetatic acid			Kim et al., 2016
	Mancozeb (Dithan)	Disturbs biochemical activities of cytoplasm and mitochondria	–	Hossain M. S. et al., 2015; Fungicide Resistance Action Committee, 2020
	Fluazinam	Restricts production of energy by deprotonating or protonating the amino acids	–	Qu et al., 2018
	Phenamacril	Interferes in activity of ATPase of fungal mayosin in domain of class I	K218T, S219L, and S219P, myosin mutations	Hou et al., 2018; Li et al., 2018; Wollenberg et al., 2019; Wu et al., 2020
	Fludioxonil (Cannonball)	Interference in fungal respiration	–	Hossain M. S. et al., 2015; US EPA, 2022
	Pydiflumetofen	Disturbs succinate dehydrogenase that inhibit energy production	–	Bai et al., 2021
	Trifloxystrobin (Flint fungicide)	Interrupt fungal respiration	–	Hossain M. S. et al., 2015
	Pyraclostrobin	Interrupt ATP production by restricting electron transportation	–	Fernández-Ortuño et al., 2012; Hossain M. S. et al., 2015
Demethylation Inhibitor	Ipconazole	Interferes in biosynthesis of ergosterol	–	Li et al., 2018
	Difenoconazole (Score)	Restricts sterol biosynthesis	–	Hossain K. S. et al., 2015
	Triflumizole (Trifmine)	Inhibits biosynthesis of ergosterol	–	Suzuki et al., 1994; Singh et al., 2019
	Penconazole (Topas)	Inhibits ergosterol synthesis	–	Kumar et al., 2020
	Pefurazoate (Healthied)	–	–	Singh et al., 2019
	Propiconazole (Protaf)	Blockage of 14-α-sterol demethylase activity	–	Gad and Pham, 2014; Hossain K. S. et al., 2015
	Prochloraz (Sportak)	–	Efflux transference, degradation of prochloraz, Alteration in cell wall structure, CYP51B and S312T mutations	Park et al., 2009; Kim et al., 2010; Yang et al., 2012a,b; Kumar et al., 2020; Zhang et al., 2021

#### 8.7.2. Sterol demethylase inhibitors

Site-specific fungicides such as sterol demethylase inhibitors (SDMIs) have been applied as an alternative to benzimidazole fungicides against bakanae disease (Table 3). These fungicides target the CYP51 enzyme i.e., P450-sterol 14α- demethylase. This enzyme is necessary to biosynthesize the ergosterol, which is a constituent of the cell membrane that fungi require to grow. Three genes of *CYP51* including *CYP51A, CYP51B*, and *CYP51C* are found in *F. fujikuroi* (Zhang et al., 2021). Demethylase inhibitors (DMIs) are chemically classified as pyrimidine, pyridine, piperazine, triazole, and imidazole. The antifungal efficacy of triazole has been assessed to control *F. moniliforme* by using 25% tebuconazole emulsifiable concentrate (EC), 25% propiconazole EC, and 2.5% difenoconazole EC (Hossain K. S. et al., 2015). Application of triazole fungicides such as propiconazole (Bagga and Sharma, 2006) and ipconazole (Tateishi and Suga, 2015; Li et al., 2018) and imidazole fungicide such as prochloraz (Kumar et al., 2020) significantly suppressed *F. fujikuroi* growth. Soaking of rice seeds for 24 h in a diluted solution of 200-folds of ipconazole wettable powder at 6% or ipconazole at 0.0472 μg/mL showed efficacy against bakanae disease. Application of 0.05% propiconazole EC on rice seedlings also effectively controlled the bakanae disease of rice; however, a side effect of its toxicity resulted in reduced plant height and crop yield (Bagga and Sharma, 2006). The application of prochloraz 10 μg/mL completely suppressed *F. fujikuroi* growth (Park et al., 2009). Suzuki et al. (1994) found that the application of triflumizole EC as a seed treatment was more effective against bakanae disease as compared to triflumizole WP application. The application of pefurazoate, which is an imidazole fungicide, 500 μg/mL as a seed treatment reduced 90% of disease incidence (Singh et al., 2019). Under *in vitro* tests, penconazole and difenoconazole fungicides showed high antifungal efficacy against *F. moniliforme* (Kumar et al., 2020). However, a study conducted under greenhouse conditions indicated that the application of difenoconazole fungicide as soil drenching effectively controlled the bakanae disease (Kumar et al., 2020).

The resistance mechanism of *F. fujikuroi* to SDMIs (upon their excessive use) is presented in Table 3. A Korean strain CF245 of *F. fujikuroi* showed resistance to prochloraz by degrading it (Kim et al., 2010). Consequently, an efflux transporter was responsible for resistance to prochloraz in this Korean strain (Kim et al., 2010). Furthermore, in another Korean strain of *F*. *fujikuroi* CF337, the alterations in fungal cell structures and changes in ATP binding cassette transporter have been revealed to play a role in prochloraz resistance (Yang et al., 2012a). In yet another study, the cause of resistance in Chinese *F. fujikuroi* against prochloraz was determined as the overexpression of *CYP51* genes and mutations S312T that occurred in CYP51B (Zhang et al., 2021). However, the link between F box or WD repeat protein and survival factor 1 with the sensitivity of *F. fujikuroi* to prochloraz fungicide was found. The resistance to prochloraz was reduced due to the disruption of genes survival factor 1 whereas, the disruption of genes in F box or WD repeat protein enhanced resistance in *F. fujikuroi* to prochloraz (Choi et al., 2017).

### 8.8. Other fungicides

In addition to benzimidazoles and SDMIs, other fungicides have also been applied against the bakanae disease of rice (Table 3). Phenamacril is a new cyanoacrylate chemical fungicide that restricts the class I myosin ATPase activity (Hou et al., 2018) and significantly inhibits the *F. fujikuroi* growth when applied at 0.1544 μg/mL (Li et al., 2018). However, some strains of the Chinese *F. fujikuroi* showed resistance to phenamacril fungicide (Hou et al., 2018). It has been indicated that the high resistance of *F. fujikuroi* to phenamacril is associated with the mutations S219L, S219P, and K218T in myosin-5 (Hou et al., 2018; Wu et al., 2020). High antifungal activity was shown to inhibit *F. fujikuroi* by using fluazinam fungicide which belonged to the class arylaminopyridine (Qu et al., 2018), and succinate-dehydrogenase inhibitor pydiflumetofen (Bai et al., 2021). Application of pydiflumetofen at 0.1-0.2 g active ingredient/kg of this fungicide as a seed treatment showed 90% inhibition of rice bakanae disease (Bai et al., 2021). Furthermore, high antifungal activity was found against *F. fujikuroi* in chelating agents, such as chitosan oligosaccharides and ethylene-diamine-tetra-acetic acid (Kim et al., 2016). The application of combined fungicides has also been studied and proven effective in controlling the bakanae disease. Under *in vitro* conditions, it has been found that 5% pyraclostrobin with 55% metiram, 63% mancozeb with 12% carbendazim, 2.5% celest extra 5 EC with 2.5% fludioxonil, and 100 g/L trifloxystrobin with 200 g/L tebuconazole completely suppressed the *F. moniliforme* mycelial growth (Hossain K. S. et al., 2015).

### 8.9. Plants and microbial extracts

The use of natural products including plants and microbes is also effective in controlling bakanae disease. Among microbial extracts, *Bacillus* sp. extract, i.e., surfactin A, has been reported to suppress *F. moniliforme* growth up to 16% upon application at 2,000 μg/mL (Sarwar et al., 2018). Extract of *P. polymyxa* in the form of crude protein restricted *F. moniliforme* activity (Khan et al., 2020). Tumescence and distortion of fungal spores were found by using crude protein extract (Zhang et al., 2010). Additionally, extracts of plants such as *Artemisia judaica, Eucalyptus globulus, Coriandrum sativum*, and *Ammi visnaga* effectively reduced *F. fujikuroi* mycelial growth (Kalboush and Hassan, 2019). Essential oils extracted from *Cinnamomum tamala* (Baria and Rakholiya, 2020)*, Eucalyptus citriodora* (Gupta and Kumar, 2020), *Cymbopogon martini* (Akhila, 2009), and *Mentha piperita* (Habibi et al., 2018) were reported to have antifungal activity and effectively suppressed *F. fujikuroi* activity. Among them, the highest antagonistic potential was found in *C. martini* var. *motia* oil (Baruah et al., 1996). Though control methods for bakanae disease of rice are generally applied before the infection occurs, silica nanoparticles, formed from the husks of rice, applied as a foliar spray after the development of bakanae disease symptoms effectively decreased the bakanae incidence (Elamawi et al., 2020).

### 8.10. Physical control

#### 8.10.1. Seed treatments

Cultivation of healthy (non-infected) seeds is very important for the prevention of bakanae disease because of its seed-borne nature. Immersion in hot water is useful to disinfect the seeds. The immersion of infected rice seeds in hot water for 10–20 min at 58–60°C has the potential of disinfection similar to traditional chemical applications (Kim et al., 2022). However, precise control of water temperature is a key factor in the successful disinfection of the seeds. Salt water can also be used to select non-infected rice seeds. Immersion in hot water and the selection of non-infected rice seeds using salt water significantly reduces the requirement for chemical fungicides. However, it is hard to completely eliminate the infected rice seeds by following these seed treatment methods. Irradiation with atmospheric plasma has also been reported to be efficient to disinfect the seeds; in addition, irradiation has reduced the disease severity of rice bakanae up to 18.1% more than without irradiation control (Ochi et al., 2017).

#### 8.10.2. Agronomic practices

Conventional agronomic strategies are eco-friendly control methods. Such practices including the application of organic fertilizer, suitable planting practices, and crop rotation can be effective against rice bakanae disease. The source of infection can be minimized by burning the crop residues and infected plants (Sunder et al., 2014; Bashyal et al., 2016). Habitat manipulation is also an effective approach to controlling several soil-borne pathogens (Shakeel et al., 2022c). To prevent bakanae, a support vector machine classifier has also been developed to differentiate the infected and non-infected rice seedlings (Chung et al., 2016). Furthermore, it has been indicated that the late sowing of seedlings also reduced the incidence of bakanae. For instance, in Australia, crop rotation of rice with meadow grass and cultivation of rice seeds in late January instead of December has been highly recommended to prevent bakanae of rice in early maturing varieties (Sunder et al., 2014).

## 9. Conclusions and future perspectives

The emergence of the bakanae disease poses a significant threat to global rice production. The global rice industry suffers massive yield losses (up to 90%) due to the bakanae disease. The pathogen is generally seed-borne in nature and can also survive in the soil. *F. fujikuroi*, one of the several species of the genus Fusarium, reported as the causal agent of bakanae disease, has been abundantly found to be associated with the disease as the most virulent species worldwide. Trichoderma, Penicillium, Pseudomonas, and Bacillus strains are some of the natural antagonists that have all been shown to be effective to control the disease. A rapid breakout in commercial rice cultivation regions may result in considerable production losses and be very challenging to control after the bakanae disease has entered the field. Therefore, it is crucial to generate genetically-resistant kinds to ensure a high harvest. Different QTLs of bakanae disease resistance have been identified. Among them, the recently identified qBK4T on chromosome 4 is involved in the regulation of rice resistance to bakanae disease. It contains genes with novel molecular activities. Understanding the involvement of these genes in the immune response to the bakanae disease may require functional characterization. This suggests that further functional investigations may shed light on the molecular activities of qBK4T-related genes and their likely relationships with other familiar defense-related genes in the response to bakanae disease. The bakanae disease has been extensively studied from several perspectives worldwide. There is a pressing need, however, for more study of the biochemical and molecular features of pathogenesis as well as racial profiling, disease mechanism, virulence pattern, and other related topics. Disease resistance, possible antagonists, and biodegradable chemical compounds, along with a decision support system require special consideration as we worked to develop effective disease management strategies. According to projections made using climate change scenarios, the prevalence of rice bakanae will either rise or at the very least stay the same in the future. It is crucial to first comprehend the epidemiological makeup of the target disease to reduce any future risk of bakanae sickness. In this study, thorough analyses of several components, including the pathogen, environment, and the features of the illness cycle, have been provided. The majority of bakanae management alternatives were also discussed in this study, and it encouraged the adoption of the most efficient combination of several choices, including chemical control, biological control, cultural control, resistive control, and physical control measures. Fungicides are only used with other methods in IDM when it is absolutely essential for more efficient and long-term control. Initially, during the land preparation for rice paddy fields, an ideal amount of fertilizer should be administered, with no particular element in excess or shortage. Also, the cultivation of rice types that are resistant to bakanae should be prioritized in the event of a bakanae epidemic in the field. By growing these types, bakanae production losses can be minimized with fewer fungicide treatments.

In recent years, research on non-destructive early detection of crop diseases using hyperspectral sensors has increased in response to the growing global interest in precision agriculture. A few difficulties in establishing the presence or absence of pathogens inside the thick seed coverings are anticipated given the nature of hyperspectral remote sensing, which evaluates the features of the target through spectral reflectance. It is anticipated that the illness may be identified using hyperspectral and thermal imaging, given that the field has an extended overgrowth, which is a characteristic indication of bakanae. However, the use of remote sensing imaging methods in plants to identify the bakanae symptoms is currently constrained, primarily due to its secondary symptoms, which include pigment, structure, fluorescence, and temperature. In addition, it might be challenging to distinguish whether the symptoms are brought on by bakanae alone because the physiological indications mentioned above can vary based on variables including the habitat and rice variety. Consequently, using a bakanae infection model to comprehend the epidemiological behavior of bakanae impacted by daily weather conditions across the rice growth stages would be a more practical option for the early identification of bakanae in the fields. The infection model may be used to mimic the emergence of seedling infection and the subsequent effects of common seedling symptoms including elongation, stunting, and withering. Based on empirical relationships identifying the many connections between weather, pathogen, and host plant, leading to infection and symptom development in rice seedlings, seedling infection algorithms may be created. Based on controlling meteorological conditions and pertinent infection algorithms that have been established from numerous *in vitro* and field research, floral infections that result in seed infection can also be predicted during the blooming stage. The bakanae infection model that we utilized in this study for assessing the effects of climate change can serve as a springboard for future research and be used for the early identification and efficient management of bakanae in rice fields.

## Author contributions

QS, MM, and RTB: writing draft, software, and figure preparations. MAS, SA, MAA, SKU, and PKD: collection literature, formal analysis, preparation of tables, and editing. YI: validation and finalization of the review. LZ: supervision, project administration, resources, and funding acquisition. All authors have reada and agreed to the published version of the manuscript, listed have made a substantial, direct, intellectual contribution to the work, and approved it for publication.
